# Preliminary evaluation of microcarrier culture for growth and monoclonal antibody production of CHO-K1 cells

**DOI:** 10.1186/1753-6561-5-S8-P111

**Published:** 2011-11-22

**Authors:** M Elisa Rodrigues, Pedro Fernandes, A Rita Costa, Mariana Henriques, Joana Azeredo, Rosário Oliveira

**Affiliations:** 1 IBB-Institute for Biotechnology and Bioengineering, Centre of Biological Engineering, University of Minho, Braga, Portugal

## Background

The large-scale production of biopharmaceuticals commonly relies on the use of suspension cell cultures, since they provide higher yields than adherent cultures. However, most mammalian cells grow in adherent mode and therefore need to go through a process of adaptation to suspended growth, which is not always simple or feasible. In this context, microcarrier cultures have introduced new possibilities, allowing the practical culture of anchorage-dependent cells, in suspension systems, achieving high yields. In these systems, cells grow as monolayers on the surface of small spheres – the microcarriers, which are usually kept in suspension in the culture medium by gentle rocking. The aim of the present study was to evaluate, compare and optimize the use of microcarrier culture for the growth and monoclonal antibody (mAb) production of CHO-K1 cells.

## Material and methods

Two types of microcarriers were assessed and compared in this study: macroporous and microporous. For this, cultures of mAb-producing CHO-K1 cells were performed in vented conical tubes, at 37 °C and 5 % CO_2_. CHO-K1 culture assessment was divided in two phases: the initial cell adhesion phase; and the cell proliferation phase. A set of different conditions was tested, namely: initial cell concentration (2x10^5^ cells/ml and 4x10^5^ cells/ml), microcarrier concentration (1 g/L for macroporous and 3 g/L for microporous), type of rocking during the initial phase of adhesion (continuous and pulse) and during the cell proliferation phase (continuous). Medium was renewed on a daily basis and the concentration and viability of cells adhered to the microcarriers were periodically assessed (hourly for the adhesion phase, and daily after that). Furthermore, samples were taken for antibody quantification by enzyme-linked immunosorbent assay (ELISA).

## Results

Concerning the phase of initial cell adhesion to the microcarriers, it was observed that cell adhesion to the microporous microcarriers is favored by the use of a higher initial cell concentration (4x10^5^ cells/ml) with both pulse and particularly continuous rocking methodologies. On the other hand, cell adhesion to the macroporous microcarriers is favored by a higher initial cell concentration, but only with continuous rocking. For a lower initial cell concentration, a pulse rocking methodology is recommended. For both microcarriers, the majority of cell adhesion occurs within the first 3 hours.

Regarding the cell proliferation phase, the results showed that it is affected by the inoculum concentration only for the microporous microcarriers, with 4x10^5^ cells/ml providing the best cell proliferation. Comparing the two types of microcarriers in terms of cell growth, it was observed that the microporous provided higher cell proliferation than the macroporous. Additionally, the microporous microcarriers demonstrated a higher durability than the macroporous, which starts to disintegrate after two weeks.

Concerning the results of mAb production, it was observed that in microporous cultures it is favored by the use of a pulse rocking methodology in the initial phase of adhesion, for both inoculum concentrations evaluated. This was also observed in macroporous cultures but only for the lowest concentration of cells. With 4x10^5^ cells/ml the use of a continuous rocking methodology proved to be advantageous. Indeed, the highest level of production and productivity was achieved in these conditions – 4x10^5^ cells/ml and continuous rocking. Furthermore, the results show that the cells have higher productivities when cultured in macroporous microcarriers than in microporous, in spite of having better levels of proliferation in the last one. Indeed, with fewer cells, the macroporous carrier was able to provide levels of total mAb production similar and even greater than the microporous.

## Conclusions

This study demonstrated that microcarrier cultures are a viable alternative to suspended cultures for the growth and antibody production of CHO-K1 cells. For this purpose, the use of higher inoculum concentrations during the initial phase of cell adhesion is particularly favorable if continuous rocking is used.

The comparison of the two different types of microcarriers assessed indicated that, in general, higher levels of cell adhesion and proliferation are obtained with microporous microcarriers, while higher mAb productivity and total production are achieved with the macroporous. Therefore, the microporous microcarriers assessed is recommended for purposes of cell growth while the macroporous is indicated for purposes of production. Among the culture conditions tested, the most favorable for the purpose of mAb production is the use of the macroporous microcarriers cultured at 4x10^5^ cells/ml under continuous rocking.

